# Central Retinal Vein Occlusion in patients with COVID-19 infection: A systematic review

**DOI:** 10.1016/j.amsu.2021.102898

**Published:** 2021-10-08

**Authors:** Irfan Ullah, Aruba Sohail, Mir Umer Farooq Alam Shah, Maman Khurshid, Mufaddal Najmuddin Diwan, Abdul Qadir, Muhammad Irfan

**Affiliations:** a Kabir Medical College, Gandhara University, Peshawar, 25000, Pakistan; b Dow University of Health Science, Karachi, Pakistan; c Internal Medicine, Khyber Medical College, Peshawar, Pakistan; d Internal Medicine, Hayatabad Medical Complex, Peshawar, Pakistan

**Keywords:** COVID-19, CRVO, Central retinal vein occlusion, Pandemic

## Abstract

This systematic review summarizes the evidence on patients diagnosed with central retinal vein occlusion (CRVO) secondary to COVID-19. We searched PubMed and Google Scholar from its inception till June 2021. From an initial 55 publications, 10 studies provided specific information on COVID-19 patients with CRVO. Studies described 10 patients, 60% were male and the mean age was 39.3 ± 11.6 years. Blurred vision (40%) and decreased vision (50%) were the most common presenting complain. Symptom onset ranged from 5 days to 6 weeks after initial complaint of fever. Laboratory results showed elevated inflammatory markers and D-dimers in 60% of patients included in our review. Common treatment options were intravitreal anti-VEGF injections, steroids, and anticoagulants. Traditional co-morbidities like diabetes mellites, hypertension, and morbid obesity (hyperlipidemia) were observed in only 3/10 patients. The prognosis was excellent as all patients saw improvement in their condition. Our findings highlight the importance of identifying CRVO as an important complication of COVID-19 infection. Thus, physicians should not overlook the likelihood of CRVO in patients with COVID-19 infection and offer prompt treatment.

## Highlights

• All published CRVO cases reported to date secondary to COVID-19 disease since the beginning of the pandemic has comprehensively been summarized.
• Timely initiation of appropriate treatment could bring about complete resolution of the disease.
• Treatment with aspirin resulted in good clinical outcomes, early anticoagulant prophylaxis could be considered in patients with systemic comorbidities and severe COVID-19 infection as they could be more susceptible to CRVO.

## 1 Introduction

COVID-19, caused by severe acute respiratory syndrome coronavirus 2 (SARS-CoV-2), has had health implications of unprecedented magnitude and continues to ravage healthcare systems across the globe. As of August 8^th^ 2021, more than 200 million cases have been reported globally resulting in more than 4 million deaths [1]. The disease begins with a mild clinical course with fever, dry cough, malaise, dyspnea and loss of smell and taste. The progression of the disease could lead up to acute respiratory distress syndrome (ARDS), septic shock, and multi-organ failure which can progress to cardiac arrest with poor survival outcome [2,3]. COVID-19 has been linked to coagulation abnormalities and a prothrombotic state. Blood hypercoagulability, elevated D-Dimer, PT and aPTT prolongation, fibrin degradation products increase are some of the findings consistently reported in COVID-19 patients [4,5]. Thus, both hospitalized and ambulatory COVID-19 patients are at high risk for venous thromboembolism [4]. The system-wide thromboembolic state could affect almost every organ of the body, including the eye. Various ophthalmic manifestations of SARS-CoV-2 have been reported, including Central Retinal Vein Occlusion (CRVO) [6]. In COVID-19, the retinal vasculature could be altered by thromboembolisms, a prohaemostatic state, hypoxia and endothelial injury, causing RVO [7]. Herein, we chronicle the relationship between COVID-19 and CRVO, collating the available sparse literature and positing future directions.

Retinal vein occlusion (RVO) is the second most common retinal vascular disease after diabetic retinopathy; CRVO being one of its types [8]. The prevalence of retinal vein occlusions in the developed world has been found to be 5.20 per 1000, and the prevalence of CRVO is 0.8 per 1000 [9]. CRVO is further divided into two categories: non-ischemic (perfused) and ischemic (non-perfused). Non-ischemic CRVO is associated with good visual acuity and mild visual changes; however, ischemic CRVO carries a poorer prognosis [8]. Classic risk factors for the development of CRVO include age (90% of patients older than age 50), hypertension, glaucoma, diabetes mellitus, and hyperlipidemia [[9], [10], [11]]. Thus, CRVO is generally considered a disease of the elderly. In younger patients, hypercoagulable state has been found to be significantly associated with CRVO; however, diabetes mellitus and hypertension were not significant [12]. Individuals with SARS-CoV-2 infection may have a number of coagulation abnormalities suggesting a hypercoagulable state, which has been called COVID-19 associated coagulopathy [13]. Thus, the hypercoagulable state due to Covid-19 could be associated with CRVO. Only 1/3rd of older CRVO patients improve without treatment and there is a possibility that non-ischemic CRVO could progress to ischemic CRVO [8]. Hence, early diagnosis and appropriate management is necessary for an improved prognosis. Work up for CRVO should include assessments of visual acuity, Rapid afferent pupillary defect, fundoscopy, optical coherence tomography (OCT) and Fluorescein angiography [14]. Currently, the most common therapy used is by blocking intraocular VEGF by injecting intravitreal anti-VEGF agents such as aflibercept, bevacizumab or ranibizumab. In addition, intraocular and systemic steroids can be used to treat macular edema as well [15]. A close follow-up is maintained until the visual acuity improves and fluid activity resolves completely. In this review, we discussed the pathophysiological mechanisms leading to CRVO secondary to COVID-19. Also, we conducted a systematic review of case reports to evaluate characteristics and clinical course of patients having CRVO secondary to COVID-19.

## 2 Pathophysiology

Anatomically, the central retinal artery shares a common sheath of adventitia with the central retinal vein, located posterior to the lamina cribrosa at the arteriovenous crossing. Classically, CRVO is induced by compression of the vein by the artery as a result of atherosclerosis of central retinal artery [8].

The Virchow's triad (hypercoagulability, endothelial damage, and stasis) plays a key role in the development of retinal vein occlusions (RVOs) as it does for other thrombotic conditions; thus, both local and systemic conditions affecting one of the three components should be considered as a risk factor for CRVO [16]. Retinal findings following infection with SARS-CoV-2 virus occur due to complement activated thrombotic microangiopathy and hypercoagulable state [17,18]. The most typical finding in patients with COVID-19 and coagulopathy is an increased D-dimer concentration, a relatively modest decrease in platelet count, and a prolongation of the prothrombin time [19]. Many different studies have shown a strong association between elevated D-dimer levels vis-à-vis severity and prognosis of disease with specific concerns about thrombotic complications of COVID-19 like pulmonary embolism, stroke, disseminated intravascular coagulation, limb, and digit infarcts [17,20]. Thus, the hypercoagulable state of COVID-19 patients has been assessed by an increase in D-dimer levels as it indicates an increased risk of thrombosis [[21], [22], [23]]. The findings indicate that Covid-19 can induce a DIC-like state, referred to by some authors as “Covid-19 associated coagulopathy (CAC)”, which increases the risk of thromboembolic complications such as CRVO [13,21,24].

In a subset of patients most severely affected by COVID-19, a cytokine storm profile can be found, characterized by high concentrations of proinflammatory cytokines and chemokines [25,26]. The hyper-inflammatory profile constitutes significant increases in fibrinogen, C-reactive protein (CRP), erythrocyte sedimentation rate (ESR), interleukin-6 (IL-6) and ferritin levels [27,28]. The cytokines storm stimulates the expression of tissue factor on monocytes/macrophages and vascular endothelial cells, on whose surfaces the coagulation cascade is initiated. The thrombus formation at the microvascular level contributes to tissue ischemia and organ dysfunction [29]. Moreover, intermittent hypoxia due to pneumonia can induce endothelial cells release of tissue factor, triggering the extrinsic coagulation pathway [30]. Thus, initiation of coagulation cascade as a result of these factors increases the risk of thrombus formation, consequently increasing the risk of CRVO.

SARS-CoV-2 binds to host cells via the angiotensin converting-enzyme (ACE) 2 receptor (R) – a metallopeptidase. The high expression of ACE2 receptors within endothelial cells raises a question of its vulnerability to SARS-CoV-2 binding, and the pathogen's ability to produce systemic endothelial dysfunction [31]. A pseudo-vasculitis state can result from viral infiltration of the endothelial cells of retinal vessels [32]. COVID-19 may primarily be a vascular disease causing severe endothelial disruption, complement activation, and generalized inflammation, leading to an overall procoagulant state [33]. Thus, disruption of retinal endothelium as a result of viral infiltration increases the risk of thrombus formation and CRVO. Moreover, endothelial cell dysfunction could also lead to breakdown in the inner blood-retinal barrier, resulting in increases capillary permeability and subsequent development of macular edema [34].

## 3 Literature review

The work has been reported in line with the PRISMA 2020 criteria [35]. We perused the PubMed and Google scholar databases using the medical subject headings (MeSH) “COVID-19”, “covid-19”, “SARS-CoV-2”, “severe acute respiratory syndrome coronavirus 2”, “2019-nCoV”, “Coronavirus disease 2019” AND “central retinal vein occlusion”, “CRVO”. Please see the supplementary file for a detailed search strategy.

### 3.1 Inclusion criteria and study selection

**Fig. 1:**
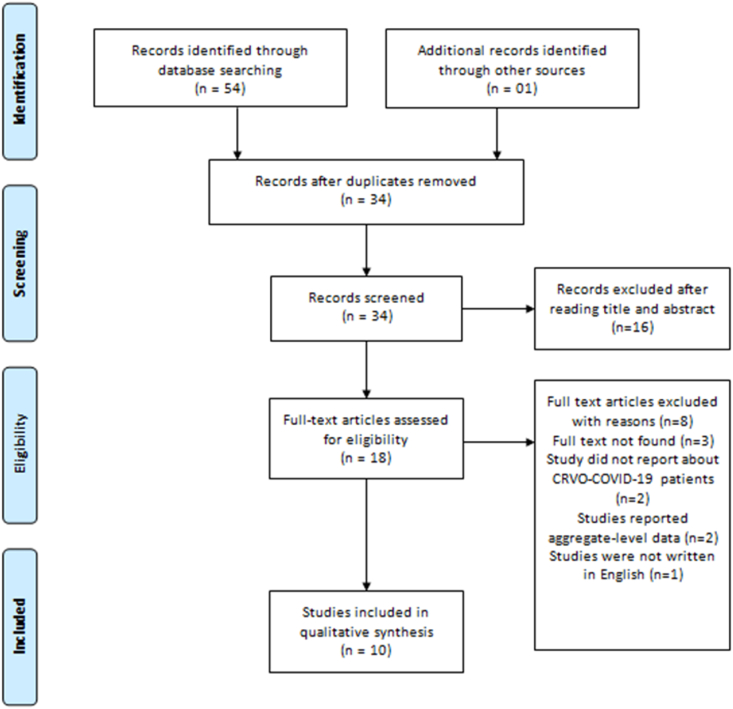
The flow chart of selected studies.

The title, abstract and full-text screening was completed in duplicate and independently by two reviewers (AS) and (MUFAS). Duplicate records were excluded, and a third reviewer (MK) was approached to resolve conflicts between authors in study selection. Articles in languages other than English were excluded. Case reports, case series, correspondence articles, and editorials were included in the present review. Studies were excluded if they did not present original empirical data, clinical data, or reported aggregate-level data (i.e., retrospective cohorts) on patients having CRVO secondary to COVID-19. The combined search strategy identified 55 potential publications on CRVO secondary to COVID-19 (Fig. 1). After screening and removing duplicate studies (n = 37), the full texts of 18 reports were sought to assess for eligibility. Of these, 2 did not report about CRVO secondary to COVID-19, 2 reported aggregate-level data, 1 was in another language and 3 did not have full-text versions available.

### 3.2 Data extraction

Data extraction was completed in duplicate by two independent reviewers, and discrepancies were resolved through feedback from a third reviewer. Data were extracted on the first author, age and sex, patient characteristics, functional tests, morphological findings, diagnosis, laboratory parameters, treatment, and prognosis.

### 3.3 Quality assessment

We conducted the quality assessment of all included studies using Joanna Briggs Institute's critical appraisal tool for case reports [36]. Each qualitative answer was converted to a numeric score. The assessment was conducted by two independent reviewers and the final score was given after resolving disagreements. A detailed quality assessment is provided in the supplementary file. The quality of our systematic review was assessed using AMSTAR 2 criteria [37]. The level of compliance with AMSTAR 2 came out to be “low”. As only case reports were included in the analyses and the number of studies was low, we couldn't conduct a meta-analysis.

### 3.4 Statistical analysis

Data were extracted and entered on excel sheets. Frequency and percentage were used to report categorical variables whilst continuous variables were reported in mean form.

## 4 Result

The search found 10 studies [[38], [39], [40], [41], [42], [43], [44], [45], [46], [47]] that met our inclusion criteria and were included in the systematic review (Table 1). All these 10 studies were case reports and reported single cases. Joanna Briggs Institute critical appraisal tools assessments score ranged from 4 to 7, with a mean score of 5.8 out of a total score of 8 (Table S1). A total of 10 cases were retrieved, comprising 6 males and 4 females [[38], [39], [40], [41], [42], [43], [44], [45], [46], [47]]. The mean age of the patients was 39.3 ± 11.6 years ranging from ages 17 to 56. Notably, patients having CRVO secondary to COVID-19 were younger than the usual age of onset of the disease. Only 3 of the 10 patients had traditional risk factors of CRVO: diabetes mellites, hypertension, and morbid obesity (hyperlipidemia) [40,42,43]. The time of presentation after the first onset of fever ranged from 5 days to 6 weeks. Associated macular edema was seen in 4/10 patients [38,42,44,45]. The two patients with diabetes mellites also had superimposed diabetic retinopathy [42,43]. A staggering 60% of the patients had elevated inflammatory markers and D-dimers [[39], [40], [41], [42], [43],^47^] The prognosis was good as all the patients improved after treatment. The treatment options included intravitreal anti-VEGF injections, steroids, and anticoagulants. Although the patient reported by Venkatesh et al. was only treated with low-dose aspirin 150 mg/day, improvement was reported at follow-up [42]. As aspirin is an anti-platelet agent, it prevents the formation of new blood clots. This reduction in hypercoagulability could favor the anti-coagulant mechanisms to dissolve clots that led to CRVO; resulting in restoration of blood flow in the retinal vessels. The findings of the literature review are depicted in Table 1.

**Table 1:** Patient information gathered from literature review of articles on individuals with CRVO secondary to COVID-19.

Author	Age and sex	patient characteristic	Functional tests	Morphological Findings	Diagnosis	Laboratory Parameters	Treatment	Prognosis
Walinjkar et al. [38]	17 y/o Female	Co-morbs:Polycystic ovaries. CP: Diminished vision since two days in RE. Onset: 21 days after COVID-19.	BCVA: 6/24 RE and 6/6 LE.	On dilated evaluation, OD showed disc swelling, splinter hemorrhages, and multiple flame-shaped and blot hemorrhages in all quadrants in the retina. OCT: neurosensory detachment and cystoid macular edema.	CRVO with macular edema.	Unremarkable	Intravitreal injection of Ranibizumab(anti-VEGF) 0.05 mL of 10 mg/mL concentration at 1-month intervals.	1 month after the second injection, RE fundus showed further resolution of signs of CRVO and macular edema appeared resolved. Her RE BCVA improved to 6/12.
Invernizzi et al. [39]	54 y/o Female	CP: Multiple violet scotomas in RE since 36 h. Onset: 7 days after COVID-19.	VA: 20/40 RE and 20/20 LE. RAPD was mildly positive in the RE.	RE Funduscopy: multiple retinal hemorrhages, increased venular tortuosity, and a diffuse fern-like retinal whitening. OCT: hyperreflectivity of the inner retinal layers. Autofluorescence highlighted the typical distribution of the retinal alteration showing perivenular hypo-autofluorescence. A delayed arteriovenous transit time was observed on FA.	Impending CRVO.	CRP, ESR, LDH, PT, aPTT, fibrinogen and D-dimer elevated.	IV Methylprednisolone 1g/day was administered till the diagnosis was made. Upon diagnosis, 60 mg/day oral Prednisolone was given and later reduced to 30 mg/day.	Her vision returned to 20/20 in both eyes and multimodal imaging revealed an almost complete regression of the retinal alteration.
Gaba et al. [40]	40 y/o Male	Co-morbs: Controlled hypertension and morbid obesity. CP: DVT in right leg. Severe dilation of RV, bilateral painless blurred vision, worse in LE. Onset: 5 days after COVID-19.	VA: 6/9 RE and 6/18 LE.	Funduscopy: bilateral dilated and tortuous veins, widespread cotton wool spots, dot and blot intraretinal hemorrhages, and optic disk edema; findings more pronounced in the left fundus. OCT: 2 parafoveal hemorrhages seen in the LE.	bilateral CRVO with optic disk edema.	Elevated ferritin, lactate dehydrogenase, D-dimer, C-reactive protein, and interlekin-6.	The patient was started on LMWH which was switched to rivaroxaban, 15 mg twice daily for 21 days, then 20 mg once daily for 3 months on discharge.	Ten days after hospital admission, he had near-normal vision.
Yahalomi et al. [41]	33 y/o Male	CP: Blurred vision with flashes of light in LE for the past month. Onset: 5 weeks after COVID-19.	Uncorrected VA: 20/20 RE and 20/25 LE. RAPD was positive in LE.	LE Funduscopy: tortuosity and dilatation of all branches of the central retinal vein, dot, blot and flame-shaped hemorrhages in all four quadrants, and optic disc edema. FA: marked delay in arteriovenous transit time, staining of dilated tortuous veins and masking by retinal hemorrhages.	unilateral non-ischemic CRVO.	Slightly abnormal fibrinogen and D-dimer	Unavailable	The retinal vascular appearance improved and complete resolution occurred.
Venkatesh et al. [42]	56 y/o Female	Co-morbs: Controlled DM (on oral hypoglycemic agents) for last 5 years. CP: decreased vision in LE for the last 1 month.	BCVA: 6/6 RE and 6/18 LE	LE funduscopy: few microaneurysms, scattered retinal haemorrhages in all four retinal quadrants, hyperaemic disc and dilated retinal vessels. OCT: cystoid macular edema, shallow neurosensory detachment and intact outer retinal layers.	non-ischemic CRVO, macular edema and mild stage of non-proliferative diabetic retinopathy	Elevated D-dimer and ESR.	Low-dose aspirin 150 mg/day	After 1 month, VA improved to 6/6 in LE.
Lorca et al. [43]	30 y/o Female	Co-morbs: MODY. CP: bilateral blurred vision and myodesopsias since discharged after COVID-19 pneumonia	BCVA: 0.7 in both eyes.	Funduscopy: bilateral dilatation of retinal veins, exudates and extensive deep blot and flame-shaped hemorrhages throughout the retinal parenchyma and some vitreous bleeding. FA: microaneurysms, dilated veins, ischemic areas and distal vasculitis.	bilateral CRVO and rapidly worsening diabetic retinopathy	Elevated CRP, fibrinogen, ferritin, platelets, D-dimer and glycated hemoglobin.	Unavailable	Unavailable
Raval et al. [44]	39 y/o Male	CP: Decreased vision and floaters in RE. Onset: 8 days after COVID-19.	VA: 20/150 RE and 20/30 LE.	RE dilated funduscopy: hyperemic optic nerve head, macular thickening, tortuous vasculature, and diffusely scattered intraretinal hemorrhages. RE FA: tortuous vasculature and vessel wall staining without neovascularization, leakage, or areas of non-perfusion. OCT: CME of RE.	CRVO with macular edema.	Un-remarkable.	anti-VEGF injections of bevacizumab.	After a series of injections, the macular edema decreased significantly and the patient's visual acuity improved to 20/30 RE
Sheth et al. [45]	52 y/o Male	CP: Decreased vision in LE. Onset: 9 days after COVID-19.	BCVA: 6/60 LE and 6/6 RE.	Funduscopy: LE inferior hemi retinal vein occlusion with superonasal branch retinal vein occlusion and macular edema. FA: dilated and tortuous retinal veins in inferior and superonasal quadrants which showed significant vessel wall staining and leakage in late phases. LE OCT: serous macular detachment and significant CME with cysts. Disorganization of retinal inner layers was also seen.	Inferior HRVO with superonasal VO and macular edema.	Un-remarkable.	oral methylprednisolone (40 mg/day) and intravitreal anti-VEGF injection	BCVA improved to 6/9 after 1 month.
Finn et al. [46]	32 y/o Male	CP: Mild blurriness in superior visual field of RE for two weeks. Sudden onset paracentral scotoma in RE one month later.	VA remained 20/20 in both eyes	RE dilated funduscopy: rare, scattered hemorrhages in the inferior hemisphere and dilated and tortuous vessels inferiorly. FA: marked delay in filling of the inferior venous circulation with late staining of those vessels in RE. RE OCT: mild thickening and increased hyperreflectivity of the outer plexiform layer nasally.	HRVO	Un-remarkable.	Unavailable	Unavailable
Insausti-Garcia et al. [47]	40 y/o Male	CP: Persistent and painless decrease in the sensitivity of vision in LE. Onset: 6 weeks after COVID-19.	In the visual field, a diffuse sensitivity decrease was observed, associated with a slight central scotoma and a moderate increase in the blind spot. One week after diagnosis, patient VA decreased to 20/200.	LE funduscopy: severe inflammation of the optic nerve head with retinal venous vasodilatation and tortuosity, cotton-wool spots and moderate superficial hemorrhages in all four quadrants. FA: discrete venous staining and leakage, in addition to leakage and late staining from the optic disc. OCT: papillary edema without involvement of the macular area.	Papillophlebitis with CRVO.	Elevated D-dimers, fibrinogen and CRP.	Before diagnosis, treatment was started with acetylsalicylic acid and bromfenac eye drops. After the diagnosis, sustained-release dexamethasone implant was intravitreally injected.	VA improved to 20/40 after 2 weeks.

RE; right eye, LE; left eye, DVT; Deep vein thrombosis, DM; diabetes mellitis, DM; diabetes mellitis, MODY; mature-onset diabetes mellitis, CRVO; Central retinal vein occlusion, OCT; Optical Coherence Test, HRVO; hemi-retinal vein occlusion, BCVA; Best corrected visual acuity, CME; cystoid Macular edema, VO; vein occlusion, VA; Visual acuity, ESR; erythrocyte sedimentation rate, CRP; C-reactive protein, Anti VEGF; anti-vascular endothelial growth factor, LMWH, low molecular weight heparin, FA: fluorescin angiography., RAPD; Rapid Afferent Pupillary Defect.

## 5 Discussion

The studies in the review describe ophthalmic exam findings and patient outcomes of CRVO secondary to COVID-19. We also attempted to evaluate the pathophysiological mechanisms that could lead to CRVO secondary to COVID-19. Classically, the patients having CRVO present with significantly reduced vision. They may exhibit an afferent pupillary defect in the affected eye and/or a reduction in visual acuity; Ophthalmic examination findings include cotton wool spots, swelling of the optic disc, extensive 4-quadrant hemorrhage, tortuosity and dilation of the retinal veins [8]. These findings have also been reported in patients having CRVO secondary to COVID-19. Thus, the findings and presenting symptoms in patients with CRVO secondary to COVID-19 are similar to those in patients having CRVO as a result of other etiologies.

Classically, the risk factors for CRVO include age (90% of patients older than age 50), hypertension, glaucoma, diabetes mellites and hyperlipidemia [[9], [10], [11]]. However, in this study, the mean age of the patients was 39.3 years which shows that patients having CRVO secondary to COVID-19 were younger than the usual age of onset of the disease. Moreover, only 3 of the 10 patients had traditional risk factors of CRVO: diabetes mellites, hypertension, and morbid obesity (hyperlipidemia). The key takeaway is clear: the risk of acquiring CRVO secondary to COVID-19 persists irrespective of the age or presence of classic risk factors. In these patients, the hypercoagulable state as a result of COVID-19 associated coagulopathy (CAC) lends a helping hand to the development of CRVO. We have described the pathophysiological mechanisms that could lead to CRVO secondary to COVID-19. These mechanisms are more significant than classic risk factors associated with CRVO in these patients.

The patient reported by Venkatesh et al. [42] improved at follow-up despite being only treated with low-dose aspirin 150 mg/day. As aspirin is an anti-platelet agent, it prevents the formation of new blood clots. This reduction in hypercoagulability could favor the anti-coagulant mechanisms to dissolve clots that lead to CRVO, resulting in restoration of blood flow in the retinal vessels. As treatment with Aspirin resulted in good clinical outcomes, early anticoagulant prophylaxis should be considered in patients with systemic comorbidities and severe COVID-19 infection as they could be more susceptible to CRVO. Venous thromboembolism (VTE) prophylaxis has been recommended for hospitalized and critically ill patients [48]. As COVID-19 associated coagulopathy (CAC) could play a significant role in CRVO, anticoagulant prophylaxis could significantly decrease the risk of CRVO. Moreover, as the thromboembolic complications of COVID-19 are well established [17,20], there is a need to devise anticoagulant prophylaxis regimens bearing in mind the ophthalmic implications as well.

Diabetic retinopathy superimposed on CRVO was also reported in diabetic COVID-19 patients; thus, further studies are warranted to establish the correlation between COVID-19 and diabetic retinopathy. Given these evolving challenges, it is essential that large scale trials are devised – not just to discern the interplay between COVID-19 and CRVO, but also other thromboembolic conditions that can cause significant morbidity and mortality.

Our study has some limitations. Most of the studies in our review are case reports in which a limited number of patients are assessed. Larger scale studies with greater number of cases and longer follow-ups are needed to reliably draw the correlation between COVID-19 and CRVO. Nevertheless, there is a conspicuous correlation between the two that cannot be discounted. Moreover, studies in languages other than English were excluded. As CRVO is not a well-known complication of COVID-19, it is likely that many cases go unreported which further limits the literature available. Our study has some limitations. Most of the studies in our review are case reports in which a limited number of patients are assessed. Larger scale studies with greater number of cases and longer follow-ups are needed to reliably draw the correlation between COVID-19 and CRVO. Nevertheless, there is a conspicuous correlation between the two that cannot be discounted. Moreover, studies in languages other than English were excluded. As CRVO is not a well-known complication of COVID-19, it is likely that many cases go unreported which further limits the literature available.

## 6 Conclusion

Our review has comprehensively summarized all published CRVO cases reported to date secondary to COVID-19 disease since the beginning of the pandemic. Although the literature is limited, this review has been successful in establishing the causality between COVID-19 and CRVO. Irrespective of the traditional risk factors, hypercoagulability associated with COVID-19 can be a factor in the development of CRVO. Clinicians must be aware of this plausible complication of COVID-19 in case they come across patients displaying signs and symptoms of CRVO. Along with necessary clinical investigations, linking these signs and symptoms with the history of COVID-19 infection could provide an aide in prompt diagnosis of CRVO. As our review reported a good prognosis, timely initiation of appropriate treatment could bring about complete resolution of the disease.

## Provenance and peer review

Not commissioned, externally peer reviewed

## Funding

None.

## Ethical approval

NA.

## Consent

NA.

## Author contributions

IU: Study concept or design.

IU, AS, MUFAS, MK, and MND: Data collection, data analysis or interpretation, writing the paper.

AQ, MI: Critical revision of the article.

## Registration of research studies

1. Name of the registry: NA.
2. Unique Identifying number or registration ID: NA.
3. Hyperlink to your specific registration (must be publicly accessible and will be checked): NA.

## Guarantor

Irfan Ullah, Kabir Medical College, Gandhara University, Peshawar, Pakistan Irfanullahecp2@gmail.com, +923340968239.

## Declaration of competing interest

None.
