# Supplementary material for: Central Retinal Vein Occlusion in patients with COVID-19 infection: A systematic review
Source: Ann Med Surg (Lond). 2021 Oct 8;71:102898. doi: 10.1016/j.amsu.2021.102898 (PMC8500694; doi:10.1016/j.amsu.2021.102898)
Supplement: Multimedia component 1 [file mmc1.docx]

**Supplementary file**

Search strategy for COVID-19 patients with Central retinal vein occlusion

**PubMed:**

1) For COVID-19

("covid 19"[All Fields] OR "covid 19"[MeSH Terms] OR "covid 19 vaccines"[All Fields] OR "covid 19 vaccines"[MeSH Terms] OR "covid 19 serotherapy"[All Fields] OR "covid 19 serotherapy"[Supplementary Concept] OR "covid 19 nucleic acid testing"[All Fields] OR "covid 19 nucleic acid testing"[MeSH Terms] OR "covid 19 serological testing"[All Fields] OR "covid 19 serological testing"[MeSH Terms] OR "covid 19 testing"[All Fields] OR "covid 19 testing"[MeSH Terms] OR "sars cov 2"[All Fields] OR "sars cov 2"[MeSH Terms] OR "severe acute respiratory syndrome coronavirus 2"[All Fields] OR "ncov"[All Fields] OR "2019 ncov"[All Fields] OR (("coronavirus"[MeSH Terms] OR "coronavirus"[All Fields] OR "cov"[All Fields])) AND ("sars cov 2"[MeSH Terms] OR "sars cov 2"[All Fields] OR "covid"[All Fields] OR "covid 19"[MeSH Terms] OR "covid 19"[All Fields]) AND ("covid 19"[All Fields] OR "covid 19"[MeSH Terms] OR "sars cov 2"[All Fields] OR "sars cov 2"[MeSH Terms] OR "severe acute respiratory syndrome coronavirus 2"[All Fields] OR "ncov"[All Fields] OR "2019 ncov"[All Fields] OR (("coronavirus"[MeSH Terms] OR "coronavirus"[All Fields] OR "cov"[All Fields]) AND ("sars cov 2"[MeSH Terms] OR "sars cov 2"[All Fields] OR "sars cov 2"[All Fields]) AND ("sars cov 2"[MeSH Terms] OR "sars cov 2"[All Fields] OR "severe acute respiratory syndrome coronavirus 2"[All Fields]) AND ("sars cov 2"[MeSH Terms] OR "sars cov 2"[All Fields] OR "2019 ncov"[All Fields]) AND ("sars cov 2"[MeSH Terms] OR "sars cov 2"[All Fields] OR "ncov"[All Fields])

AND

2) For central retinal vein occlusion

("retinal vein occlusion"[MeSH Terms] OR ("retinal"[All Fields] AND "vein"[All Fields] AND "occlusion"[All Fields]) OR "retinal vein occlusion"[All Fields] OR ("central"[All Fields] AND "retinal"[All Fields] AND "vein"[All Fields] AND "occlusion"[All Fields]) OR "central retinal vein occlusion"[All Fields] OR "CRVO"[All Fields])

3) ONLY HUMANS

**Supplementary Table 1**

The Joanna Briggs Institute Critical Appraisal tools for use in JBI Systematic Reviews Checklist for Case Reports

| Study author | Q1 | Q2 | Q3 | Q4 | Q5 | Q6 | Q7 | Q8 | Total |
| --- | --- | --- | --- | --- | --- | --- | --- | --- | --- |
| Walinjkar et al. [38] | yes | yes | yes | yes | no | yes | no | yes | 6/8 |
| Invernizzi et al.  [39] | yes | no | yes | no | no | yes | no | yes | 4/8 |
| Gaba et al. [40] | yes | yes | yes | yes | no | yes | no | yes | 6/8 |
| Yahalomi et al.  [41] | yes | yes | yes | yes | no | yes | no | yes | 6/8 |
| Venkatesh et al.  [42] | yes | yes | yes | yes | yes | yes | no | yes | 7/8 |
| Lorca et al. [43] | yes | yes | yes | yes | no | no | no | yes | 5/8 |
| Raval et al. [44] | yes | yes | yes | yes | yes | yes | no | yes | 7/8 |
| Sheth et al. [45] | yes | no | yes | yes | yes | yes | no | yes | 6/8 |
| Finn et al. [46] | yes | yes | yes | yes | no | no | no | yes | 5/8 |
| Insausti-Garcia et al. [47] | yes | no | yes | yes | yes | yes | no | yes | 6/8 |
